# Biological and Physicochemical Analysis of Sr-Doped Hydroxyapatite/Chitosan Composite Layers

**DOI:** 10.3390/polym16131922

**Published:** 2024-07-05

**Authors:** Maria Elena Zarif, Bogdan Bita, Sasa Alexandra Yehia-Alexe, Irina Negut, Gratiela Gradisteanu Pircalabioru, Ecaterina Andronescu, Andreea Groza

**Affiliations:** 1 National Institute for Lasers, Plasma and Radiation Physics, 077125 Măgurele, Romania; maria.zarif@inflpr.ro (M.E.Z.); bogdan.bita@inflpr.ro (B.B.); sasa.yehia@inflpr.ro (S.A.Y.-A.); negut.irina@inflpr.ro (I.N.); 2 Faculty of Chemical Engineering and Biotechnologies, University Politehnica of Bucharest, 011061 Bucharest, Romania; ecaterina.andronescu@upb.ro; 3 Faculty of Physics, University of Bucharest, 077125 Măgurele, Romania; 4 eBio-Hub Research Center, University Politehnica of Bucharest-CAMPUS, 6 Iuliu Maniu Boulevard, 061344 Bucharest, Romania; gratiela.gradisteanu@icub.unibuc.ro; 5 Research Institute of the University of Bucharest (ICUB), University of Bucharest, 050657 Bucharest, Romania; 6 Department of Microbiology and Immunology, Faculty of Biology, University of Bucharest, 050657 Bucharest, Romania; 7 Academy of Romanian Scientists, 3 Ilfov Str., District 5, 050044 Bucharest, Romania; 8 National Research Center for Micro and Nanomaterials, University Politehnica of Bucharest, 060042 Bucharest, Romania

**Keywords:** hydroxyapatite, strontium-doped hydroxyapatite, chitosan, radio-frequency magnetron sputtering deposition, matrix assisted pulsed laser evaporation deposition, MTT assay, LDH assay, L929 cell line, “live/dead” cell assay, antimicrobial activity

## Abstract

In this work results are presented on the evaluation of HAp, HApSr, HAp_CS, and HApSr_CS layers deposited on Ti substrates regarding L929 cell viability and cytotoxicity as well as antimicrobial activity against *Staphylococcus aureus*, in connection with their physicochemical properties. The HAp and HApSr layers generated by radio-frequency magnetron sputtering technique were further covered with chitosan by a matrix-assisted pulsed laser evaporation technique. During the plasma depositions, the Ti substrates were heated externally by a home-made oven above 100 °C. The HApSr_CS layers generated on the unpolished Ti substrates at 100 °C and 400 °C showed the highest biocompatibility properties and antimicrobial activity against *Staphylococcus aureus*. The morphology of the layer surfaces, revealed by scanning electron microscopy, is dependent on substrate temperature and substrate surface roughness. The optically polished surfaces of Ti substrates revealed grain-like and microchannel structure morphologies of the layers deposited at 25 °C substrate temperature and 400 °C, respectively. Chitosan has no major influence on HAp and HApSr layer surface morphologies. X-ray photoelectron spectroscopy indicated the presence of Ca 2p_3/2_ peak characteristic of the HAp structure even in the case of the HApSr_CS samples generated at a 400 °C substrate temperature. Fourier transform infrared spectroscopy investigations showed shifts in the wavenumber positions of the P-O absorption bands as a function of Sr or chitosan presence in the HAp layers generated at 25, 100, and 400 °C substrate temperatures.

## 1. Introduction

The interest for calcium phosphates (CaPs) has increased over the years due to their potential in the bone tissue engineering field, namely for orthopedic and dentistry applications [1]. The production of CaPs in various physical forms [1] such as scaffolds [2,3,4], granules [5,6], or coatings [7,8,9] have been intensively studied, with a great focus on the research of composite materials. The implant failure incidence seems to be improved when implant-bone fixation is achieved using CaP composite coatings [10]. In addition to the main constituents, Ca^2+^ and PO_4_^3−^, several ions such as: Na^+^, K^+^, Mg^2+^, Sr^2+^, and Zn^2+^ can be found in bones, enamel, and dentine [11]. In order to mimic the biological and physicochemical properties of the bone, there have been multiple studies on the generation of CaP coatings doped with the above-mentioned ions [11,12,13]. Among them, Sr^2+^ is of great interest in bone tissue engineering applications due to its effects on osteoblasts, osteoclasts, and fibroblast cells. This ion improves the adhesion, proliferation, and differentiation of osteoblast and fibroblast cells, inhibiting apoptosis and bone resorption activity of osteoclast cells [14].

Plasma techniques are well known for their advantages when used for depositions of CaP compounds. Among them, the radio frequency magnetron sputtering (RF-MS) technique promotes the generation of coatings with high uniformity and adhesion to the substrate [15,16,17]. In our previous work [18], the CaP layers uniformity and adhesion to the optically polished Ti substrate was proved.

The deposition of CaP layers by RF-MS have been conducted by varying deposition parameters such as the following: electrical substrate bias, deposition time, working gas/mixture and their pressure, RF power, inter-electrode distance [19], sputtering target geometry [20] and number [21]. Rarer studies on the deposition of Sr-doped CaPs by RF-MS have been reported [22,23,24,25,26]. Moreover, in the scientific literature, there are few results on the influence of the substrate temperature on the CaP coating properties when an additional and external heat source, other than plasma, is used [19,27,28]. To our knowledge, the influence of the substrate temperature during RF-MS deposition duration on the biological and physicochemical properties of the CaPSr layers deposited on optically polished and unpolished Ti substrates has not been studied.

Chitosan (CS) is a biopolymer obtained from chitin through deacetylation [29]. Among the biomedical applications of CS, bone tissue engineering has gained increased attention in recent years. Several physical forms of CS such as sponges, beads, fibers, and films have been studied [30].

Among the deposition techniques of polymer coatings, matrix-assisted pulsed laser evaporation (MAPLE) is of great interest. During the MAPLE deposition, a frozen target, composed out of a polymeric solution, is evaporated by laser ablation. The polymer damage is minimal due to the fact that the laser energy is mainly absorbed by the solvent, which is further pumped out of the system and the material of interest reaches the substrate [31,32].

Composites based on CaPs and CS with applications in bone tissue engineering in forms of scaffolds [33], beads [34], microspheres [35,36], membranes [37,38], and coatings [39] have been reported. There are several deposition techniques used for such purpose such as: electrochemical deposition [40], dip-coating [41], micro-arc oxidation [42], galvanic deposition [43], RF-MS [39], or MAPLE [18,44,45]. The biocompatibility and antimicrobial activity of CaP_CS composite compounds was previously reported by Mina et al. [46] and by Visan et al. [45]. L929 fibroblast cells are used for biocompatibility tests due to their predisposition to death in toxic environments generated by the materials under investigation [46].

In the present research study, the relationship between the experimental deposition conditions of hydroxyapatite (HAp), strontium-doped HAp (HApSr), HAp_CS, and HApSr_CS on rough and optically polished Ti substrates and their physicochemical, and biological properties was investigated. The HAp and HApSr layers were synthesized in plasma by RF-MS technique and chitosan was further added by MAPLE technique. We were mainly focused on the effect of the external heating of the Ti substrates between 100–400 °C during the RF-MS deposition on the biocompatibility and antimicrobial properties against *Staphylococcus aureus* of the HAp, HApSr, HAp_CS, and HApSr_CS layers. Previous biological studies regarding the viability and cytotoxicity of L929 cells and antimicrobial activity against *Staphylococcus aureus*, of HApSr_CS layers generated by RF-MS and MAPLE have not been reported. The physicochemical characteristics of the layers were performed by energy dispersive X-ray spectroscopy, Fourier transform infrared spectroscopy, and X-ray photoelectron spectroscopy. By scanning electron microscopy and roughness measurements, the morphology of the layer surfaces and the influence of substrate roughness on the layer growth was revealed.

## 2. Materials and Methods

### 2.1. Materials

Titanium samples (with mirror-like and unpolished surfaces) were used as substrates (10 × 10 mm^2^).

For the synthesis of HAp and HApSr, the precursors for Ca^2+^, PO_4_^3−^, and Sr^2+^ were calcium nitrate tetrahydrate (Sigma-Aldrich; St. Louis, MO, USA; CAS number: 13477-34-4), diammonium hydrogen phosphate (Fluka; Charlotte, NC, USA; CAS number: 7783-28-0), and strontium nitrate (CAS number 10042-76-9), respectively. The pH of the solutions was adjusted using ammonium hydroxide (Sigma Aldrich; St. Louis, MO, USA; CAS number 1336-21-6).

The MAPLE targets were obtained using a low molecular weight chitosan (50,000–190,000 Da) with a 75–85% deacetylation degree, purchased from Sigma-Aldrich (CAS number: 9012-76-4).

### 2.2. Synthesis Technique

#### Microwave-Assisted Hydrothermal Synthesis

For the microwave-assisted hydrothermal synthesis of HAp, two aqueous solutions were obtained from the Ca^2+^ and PO_4_^3−^ precursors. Calcium nitrate tetrahydrate [Ca(NO_3_)_2_⋅4H_2_O] and diammonium hydrogen phosphate [(NH_4_)_2_HPO_4_] were dissolved in distilled water, under continuous stirring. The amount of the precursors was calculated in order to keep the Ca/P molar ratio equal to 1.67. A peristaltic pump (50 rpm) was used to add the (NH_4_)_2_HPO_4_ solution to the Ca(NO_3_)_2_ solution, under continuous stirring. Ammonium hydroxide (NH_4_OH) was used to adjust the pH of the final solution to 10.

The HApSr was synthesized by dissolving the proper amount of strontium nitrate [Sr(NO_3_)_2_] in the Ca(NO_3_)_2_ aqueous solution and the (NH_4_)_2_HPO_4_ in distilled water in order to maintain the (Ca + Sr)/P molar ratio equal at 1.67 and the Sr/(Ca + Sr) molar ratio equal at 0.03 [Ca_9.7_Sr_0.3_(PO_4_)_6_(OH)_2_]. The next steps of the synthesis were the same as those described for the synthesis of HAp.

The hydroxyapatite (HAp) hexagonal unit cell (space group P6_3_/m) has 44 atoms: 10 Ca atoms, 6 P atoms, 26 O atoms, and 2 H atoms. Two types of Ca atoms can be identified: Ca1 (4 atoms/unit cell) and Ca2 (6 atoms/unit cell) [47]. Each Ca1 atom is surrounded by 9 oxygen atoms: 3 O1 atoms, 3 O2 atoms, and 3 O3 atoms, while each Ca2 atom is surrounded by 7 oxygen atoms: 1 O1 atom, 1 O2 atom, 4 O3 atoms, and one OH^−^ ion [44,48]. The chemical formula of Sr-doped HAp (HApSr) is Ca_(10-x)_Sr_x_(PO_4_)_6_(OH)_2_, where 0 < x ≤ 10. Due to the small difference between the ionic radius of Ca^2+^ and Sr^2+^—1.00 Å and 1.18 Å, respectively—Sr^2+^ can enter into both Ca1 and Ca2 sites, with a preferential occupation for Ca1 site at 1–3.5 at% Sr atomic concentration and for the Ca2 site at concentrations higher than 5 at% Sr atomic concentration [49,50]. Terra et al. [49] presented the structural modifications such as lattice parameters and interatom distances determined by Sr doping of HAp at different at%, based on the substitution sites. For example, for 1%, respectively, 5% Sr in HAp the lattice parameters are a = 9.4347, c = 6.8893 and a = 9.4520, c = 6.9041 [49].

**Figure 1:**
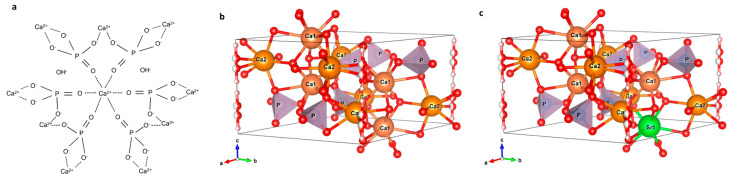
The molecular structure of HAp (**a**) and the crystalline structures of HAp (**b**) and HApSr with Sr substitution at Ca1 site (**c**); oxygen with red and hydrogen with pink.

The molecular and crystalline structures of HAp and HApSr are presented in Figure 1.

The Milestone synthWAVE microwave system (Milestone Srl, Sorisole, Bergamo, Italy) was used for the hydrothermal synthesis of HAp and HApSr. The synthesis parameters were previously described in [44] (pressure: 16 bar, temperature: 200 °C, time: 45 min–15 min heating at the desired temperature and 30 min maintained at 200 °C). The obtained precipitates were washed with distilled water and dried at 50 °C.

The HAp and HApSr RF-MS targets (4 mm thickness and 5 cm diameter) were obtained by mechanically pressing the dried powders.

### 2.3. Deposition Techniques

#### 2.3.1. Radio-Frequency Magnetron Sputtering Deposition

**Figure 2:**
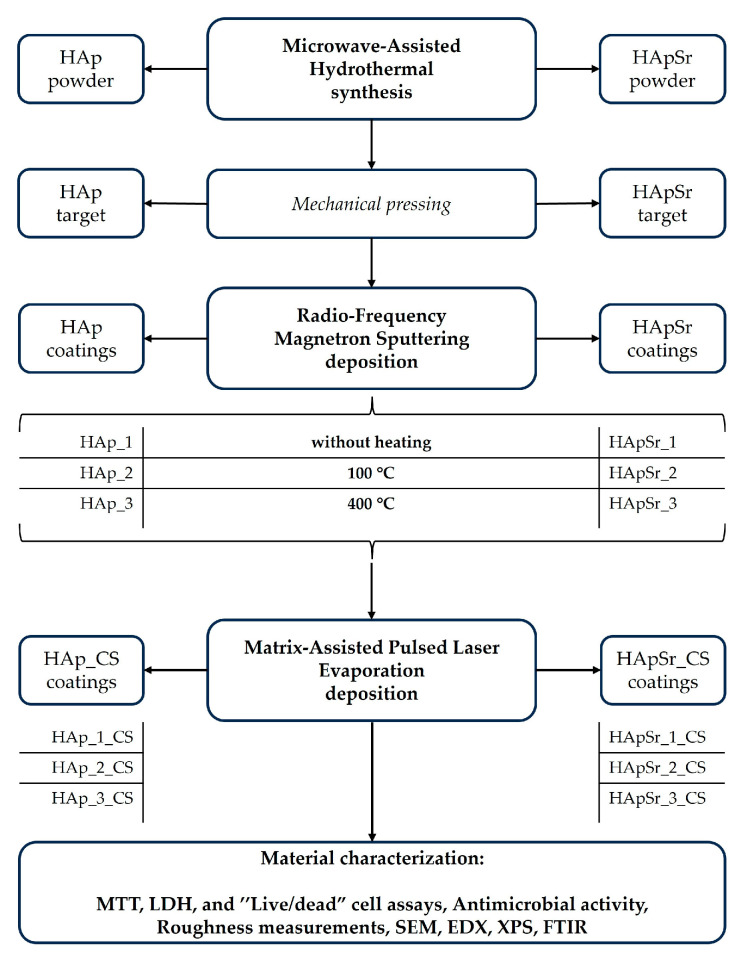
Diagram of the main methodology stages.

The HAp and HApSr layers were deposited on Ti substrates (heated from room temperature—25 °C to 100 °C and 400 °C) using the RF-MS technique. The magnetron plasma source was purchased from K.J. Lesker Company (Jefferson Hills, PA, USA). The deposition parameters and the experimental setup were reported in [44] and [18], respectively. The layer thicknesses were around 300 nm [44]. The samples codes, based on the substrate temperature and Sr content, are presented in Figure 2.

#### 2.3.2. Matrix-Assisted Pulsed Laser Evaporation Deposition

The CS layers were deposited on the HAp and HApSr layers by MAPLE technique using a KrF laser source, model COMPexPro205 Lambda Physics-Coherent (Coherent, Santa Clara, CA, USA), which works at λ = 248 nm and τFWHM = 25 ns. The deposition parameters and the experimental setup were reported in [44] and [18], respectively. The samples codes, based on CS content, are presented in Figure 2.

### 2.4. Characterization Techniques

The surface chemistry of the layers was analyzed using a K-Alpha Thermo Scientific (ESCALAB™ XI, Waltham, MA, USA) X-ray photoelectron spectrometer (XPS) with a monochromatic Al Kα X-ray source (1486.68 eV) and an X-ray spot of 900 μm diameter. The charge correction was ensured by calibrating all spectra to the main line of C 1s at 284.6 eV. The survey and high-resolution spectra were acquired with the energy step sizes of 1 and 0.1 eV, respectively. MagicPlotPro software (version 2.9) was used for XPS peak fitting analyses by a nonlinear least squares algorithm using a Gaussian curve profile [18]. The high-resolution XPS spectra of Ca 2p, P 2p, O 1s, and C 1s were fitted for all the analyzed samples under the following conditions: the area ratio for Ca 2p and P 2p was set to 1:2 for the two spin orbit peaks of Ca 2p_1/2_:Ca 2p_3/2_ and of P 2p_1/2_:P 2p_3/2_ [51], with a peak separation of ∆(Ca 2p_3/2_ − Ca 2p_1/2_) = 3.5 eV and ∆(P 2p_3/2_ − P 2p_1/2_) = 0.84 eV [18]. The peak fitting for the C 1s spectrum of each sample was conducted considering the following parameters: the FWHM was constrained between 0.7 and 1.5 eV for the peaks corresponding to C-C/C-H (alkyl type carbon), C-OH/C-O-C, and C=O, except the peak corresponding to O-C=O. The binding energy range was also constrained as follows: C-OH, C-O-C between 1.3 and 1.7 eV above the main peak (C-C/C-H), C=O between 2.8 and 3.0 eV above the main peak, and O-C=O between 3.8 and 4.3 eV above the main peak [52]. The molecular structure of the layers was identified using a Perkin-Elmer SP 100 FTIR spectrometer (Waltham, MA, USA) equipped with an attenuated total reflection unit. The Fourier transform infrared spectroscopy (FTIR) spectra were acquired with a resolution of 4 cm^−1^ and 4 scans in the wavenumber range of 4000–400 cm^−1^.

Scanning electron microscopy (SEM; ThermoFisher Apreo S scanning electron microscope, Waltham, MA, USA) and energy dispersive X-ray spectroscopy (EDX; SiLi detector) working at 1.3 × 10^−3^ Pa and 8 kV were employed to evaluate the surface morphology and the elemental composition of the layers.

Roughness measurements of the Ti substrates and of the coating’s surfaces were performed using a Mahr Perthometer S2 (Göttingen, Germany). The arithmetic mean deviation Ra of roughness profile was measured for all the analyzed samples. The optically polished Ti substrate had R_a_ = 37 nm and the unpolished Ti substrate had R_a_ = 396 nm.

The biocompatibility of the compounds was evaluated on L929 cells, cultivated in StemMACS MSC expansion media (Miltenyi Biotec, Bologna, Italy). The cells were seeded at a density of 1 × 10^5^ cells/well in 250 µL of culture medium. The cells were incubated at 37 °C (5% CO_2_) for 24 h. The evaluation of the biocompatibility was performed using the MTT, LDH, and live/dead tests.

The MTT test is a viability assay that allows the quantitative evaluation of living cells in the culture. The MTT compound [3-(4,5-dimethylthiazol-2-yl)-2,5-diphenyltetrazolium bromide] is permeable to the membranes of living cells. After 24 h of incubation, a 1 mg/mL MTT solution (Vybrant MTT Cell proliferation, Thermo Scientific, Waltham, MA, USA) was prepared and each sample was incubated in the presence of 1 mL of MTT solution for 4 h at 37 °C and 5% CO_2_. After metabolizing the MTT compound, formazan crystals were formed. These crystals were solubilized in 100 µL isopropanol. The resulting solution (violet colored) had an optical density that could be read at 550 nm (Thermo Scientific SkanIt instrument). The intensity of the color was directly proportional to the number of living cells in the sample.

Lactate dehydrogenase (LDH) is an oxidoreductase (E.C. 1.1.1.27) that is present in most organisms. Cells that no longer have membrane integrity release in the culture medium their cytoplasm that contains the LDH enzyme. It is a quantitative test that indicates the number of dead cells. The solution resulting from the reaction can be read spectrophotometrically at 490 nm (Flex Station 3). LDH (Cytotoxicity Detection kit, Sigma-Aldrich, St. Louis, MO, USA) quantification was as follows: (i) 100 µL reaction mix was prepared which contained equally all the components of the mix; (ii) 50 µL of medium were collected in duplicate from the test plate and transferred to a 96-well plate; (iii) after adding 100 µL over each sample, the plate was incubated for 15–20 min in the dark; (iv) based on the LDH level in the culture medium, the color (pink) intensity of the solution varied directly proportional to the number of dead cells.

The live/dead assay (Thermo scientific) was used to evaluate cell viability. The kit contains Calcein-AM—a non-fluorescent dye that can enter live cells where it is hydrolyzed by intracellular esterases into a green-fluorescent product and ethidium bromide—which is impermeable to live cells but can penetrate dead cells with compromised membranes, binding to nucleic acids and emitting red fluorescence. By staining cells with these dyes, live and dead cells can be distinguished using fluorescence microscopy. Cell fluorescence was measured on a Zeiss AxioScope microscope (Jena, Germany).

From an overnight culture of *S. aureus* ATCC strain, a fresh inoculum was prepared and incubated at 37 °C with shaking until a mid-exponential phase was obtained (10^8^ CFU mL^−1^). The bacterial suspension was diluted to reach a bacterial concentration of 10^6^ CFU mL^−1^. The bacterial suspension was placed into 5 mL screw-cap test tubes and incubated in the presence of the materials in a final volume of 2 mL at 37 °C with shaking. *S. aureus* ATCC was counted by taking 50 µL aliquots after 24 h of incubation making serial dilutions, and plating on plate count agar.

The methodology for the layers deposition and characterization is presented in Figure 2.

## 3. Results

### 3.1. X-ray Photoelectron Spectroscopy

The surface chemistry of the HAp, HApSr, HAp_CS, and HApSr_CS layers was evaluated by XPS.

#### 3.1.1. Hydroxyapatite and Strontium-Doped Hydroxyapatite Coatings

The XPS survey spectra of HAp and HApSr layers deposited at different substrate temperatures by RF-MS technique are presented in Figure S1. The presence of the following chemical elements: Ca, P, and O in the XPS survey spectra was identified.

To evaluate the chemical structure of the coatings, high-resolution XPS spectra (Ca 2p, P 2p, O 1s, C 1s, Sr 3d, and Ti 2p) were acquired. The high-resolution XPS spectra of Sr 3d is not shown as it overlaps with the P 2p spectra [53].

**Figure 3:**
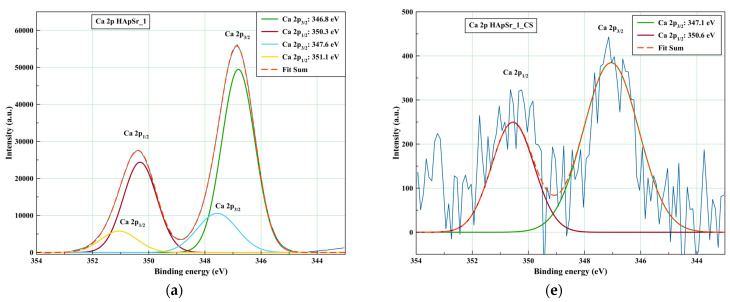

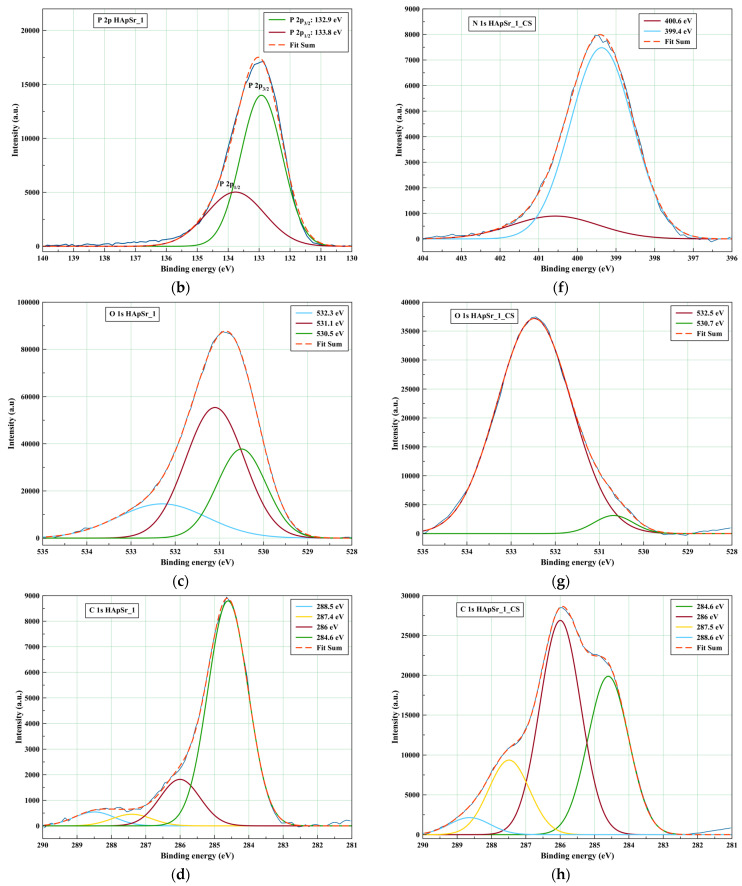
XPS high-resolution deconvoluted spectra of HAp_Sr_1: (**a**) Ca 2p, (**b**) P 2p, (**c**) O 1s, and (**d**) C 1s and HApSr_1_CS: (**e**) Ca 2p, (**f**) N 1s, (**g**) O 1s, and (**h**) C 1s.

The deconvoluted high-resolution XPS spectra of HA_Pp_1 and HApSr_1 are presented in Figure S2 and Figure 3, respectively. For the HAp_1 coating, the XPS peaks corresponding to Ca 2p_3/2_ (see Table 1 and Figure S2a), 348 eV and 347.1 eV, were assigned to Ca-O in a calcium phosphate phase [20] and Ca-Ca, Ca-OH, and Ca-O in HAp [54], respectively. The peak corresponding to P 2p_3/2_ (133.2 eV) was assigned to P-O bindings in PO_4_ of HAp (see Table 1 and Figure S2b).

For the HApSr_1 coating, the XPS peaks corresponding to Ca 2p_3/2_ (see Table 1 and Figure 3a), 347.6 eV and 346.8 eV, were assigned to the Ca-O binding energy from a calcium phosphate phase [20] and to Ca-O bonds in HAp, CaCO_3_ [55,56], respectively. The calcium carbonate binding may result due to carbon species being adsorbed at the coating surface from the air [55]. The XPS peak corresponding to P 2p_3/2_ (132.9 eV) was assigned to P-O bindings in PO_4_ of HAp (see Table 1 and Figure 3b).

For the HAp_1 coating, the peaks corresponding to O 1s (see Table 1 and Figure S2c), 532.2 eV, 531.3 eV, and 530.7 eV, were assigned to the following: HPO_4_^2−^ [18]; O-P in PO_4_^3−^, O-H in OH^−^ and O-C in CO_3_^2−^ [20]; O-Ca in HAp and surface adsorbed CaCO_3_ [20,54].

For the HApSr coating deposited without heating the substrate, the O 1s XPS peaks shifted to lower binding energies, namely 531.1 eV and 530.5 eV (see Table 1 and Figure 3c). The presence of HPO_4_^2−^ was also identified for the HApSr_1 coating at a binding energy of 532.3 eV.

For the C 1s spectra of HAp and HApSr coatings, four peaks were identified after deconvolution (see Table 1). In the case of HAp_1 (see Table 1 and Figure S2d), the peaks were identified at the following binding energies: 284.6 eV (C-C, C-H), 286 eV (C-OH, C-O-C), 287.4 eV (C=O), and 288.6 eV (O-C=O) [52]. The binding energies for the four peaks corresponding to HApSr_1 were the same (see Table 1 and Figure 3d). The binding energies of 288.6 eV and 288.5 eV may also be associated with carbonate species, due to the overlapping with O-C=O. However, carbonate species are not usually associated with adventitious carbon [52].

The XPS binding energies corresponding to the HAp and HApSr coatings deposited by heating the substrate at 100 °C and 400 °C are presented in Table 1. The XPS peaks corresponding to Ca 2p, P 2p, O 1s, and C 1s present shifts in their binding energies, indicating that the chemical structure of the coatings is influenced by the substrate temperature.

#### 3.1.2. Hydroxyapatite/Chitosan and Strontium-Doped Hydroxyapatite/Chitosan Coatings

The Ca 2p and P 2p peaks were not identified in the XPS survey spectra of the HAp_CS composite layers (Figure S3). This and the presence of chitosan characteristic peaks (O 1s, N1s, and C 1s) confirm that the HAp layers were covered by chitosan during the MAPLE deposition.

High-resolution XPS spectra were acquired for Ca 2p, P 2p, O 1s, C 1s, and N1s. The XPS peaks characteristic for Ca 2p were identified in all the high resolution XPS spectra of the CS containing layers (see Table 2), regardless of the deposition temperature and of the Sr content. No P 2p XPS peaks were recorded. This finding may suggest that CS is partially embedded in the HAp and HApSr layers [18].

The deconvoluted high-resolution XPS spectra of HAp_1_CS and HApSr_1_CS samples are presented in Figure S2 and Figure 3, respectively. For the HAp_1_CS (Figure S2e) and HApSr_1_CS (Figure 3e) coatings, the XPS peaks corresponding to Ca 2p_3/2_ (see Table 2), 347 eV and 347.1 eV, respectively, were assigned to Ca-Ca, Ca-OH, Ca-O, in HAp [20,54].

For the HAp_1_CS coating, the peaks corresponding to O 1s (see Table 2 and Figure S2g), were assigned to the polysaccharide backbone of chitosan (532.4 eV) [18] and O-Ca in HAp and CaCO_3_ [20,54] or to amide of acetylated functions (530.6 eV) [59], obtained for the HApSr_1_CS coating (see Figure 3g).

For the C 1 s spectra of HAp_CS and HApSr_CS coatings, four peaks were identified after fitting procedure (see Table 2). In the case of HAP_1_CS (see Table 2 and Figure S2h, the peaks were identified at the following binding energies: 284.6 eV (C-C, C-H [52,57,58]), 286 eV (C-OH, C-O-C [52], and C-N [59,60,61]), 287.4 eV (N-C=O [59,60,61] and C=O [52]), and 288.5 eV (O-C=O/CO_3_^2−^ [52]). The same binding energies were identified for the HApSr_1_CS coating (see Table 2 and Figure 3h). In this case, only the binding energy corresponding to O-C=O/CO_3_^2^ was shifted with 0.1 eV. These results illustrate the presence of CS on the HAp and HApSr coating surfaces.

The area percentages for the peak characteristic to C-OH; C-O-C/C-N (286 eV) (see Table 2) ranged between 43 to 46% for all chitosan containing coatings (HAp_CS and HApSr_CS), while for the coatings without chitosan (HAp and HApSr) for the peaks characteristic to C-OH; C-O-C (286 eV) (see Table 1), the area percentages were below 23% for all samples.

For the peak characteristic to N–C=O/C=O (~287.4 eV) (see Table 2) the peak area percentages were 16% for all chitosan-containing samples, while for the HAp and HApSr coatings the peak characteristic to C=O (287.4 eV) represented maximum 5% of the total area (see Table 1). It is difficult to distinguish between these peaks as the chemical shifts of C=O and N-C=O bonds relative to the C-C and C-H bonds are 2.90 eV and 3.11 eV, respectively [62].

By comparing the area percentages (from the total area) of the C-OH, C-O-C/C-N, and N–C=O/C=O peaks for the HAp/HApSr and HAp_CS/HApSr_CS coatings, it is clear evidence of the chitosan contribution.

For the N1s spectra of HAp_CS_1 (see Figure S2f) and HApSr_CS_1 (see Figure 3f), two peaks were identified at 400.4 eV and 399.3 eV and 400.6 eV and 399.4 eV, respectively, corresponding to C-NH_2_ and C–NH–C=O binding energies.

The binding energies of the deconvoluted peaks and their assignments are presented in Table 2.

**Table 2:** XPS binding energies (eV) assignment for the HAp_CS and HApSr_CS coatings.

XPS Peaks	Binding Energy (eV)						XPS Peak Assignment
	HAp_1_CS (eV)	HAp_2_CS (eV)	HAp_3_CS (eV)	HApSr_1_CS (eV)	HApSr_2_CS (eV)	HApSr_3_CS (eV)	
Ca 2p_1/2_	350.5	350.5	350.5	350.6	350.6	350.5	Ca-Ca; Ca-OH; Ca-O in HAp [20,54]
Ca 2p_3/2_	347	347	347	347.1	347.1	347	Ca-Ca; Ca-OH; Ca-O in HAp [20,54]
O 1s	532.4	532.4	532.5	532.5	532.5	532.4	Polysaccharide [18,59] backbone of chitosan
	530.6	530.6	530.7	530.7	530.6	530.6	O-Ca in HAp and CaCO_3_/amide of acetylated functions [20,54]/[59]
C 1s	288.5	288.8	288.7	288.6	288.6	288.5	O-C=O/CO_3_^2−^ [52]
	287.4	287.6	287.5	287.5	287.4	287.4	N–C=O/C=O [59,60,61]/[52]
	286	286	286	286	286	286	C-OH; C-O-C/C-N [52]/[59,60,61]
	284.6	284.6	284.6	284.6	284.6	284.6	C-C; C-H [52,57,58]
N1s	400.4	400.5	400.5	400.6	400.6	400.7	C–NH–C=O [63]
	399.3	399.3	399.4	399.4	399.4	399.4	C-NH_2_ [63]

### 3.2. Fourier Transform Infrared Spectroscopy

The FTIR spectra of the deposited films were acquired in order to identify the absorption bands characteristic of the functional groups of HAp, namely phosphate (PO_4_^3−^), hydroxyl (OH^−^), and carbonate (CO_3_^2−^) ions. In non-stochiometric HAp, the hydrogen phosphate group (HPO_4_^2−^) may also appear [18].

The phosphate ions can be identified in the FTIR spectrum based on the following vibrations: ν_3_ (asymmetric stretching mode), ν_1_ (symmetric stretching mode), ν_4_ (bending mode), and ν_2_ (bending mode), at specific wavenumbers: 1120–1000 cm^−1^, ~960 cm^−1^, a double band in the wavenumber range 600–550 cm^−1^, and ~470 cm^−1^, respectively. The hydroxyl group is evidenced in the FTIR spectrum by the stretching mode of lattice water, the bending mode of adsorbed water, and the libration mode (ν_L_), at ~3570 cm^−1^, ~1640 cm^−1^, and ~631 cm^−1^, respectively. The carbonate ion presence in the HAp structure is highlighted by absorption bands around 1600–1400 cm^−1^ and 875 cm^−1^. Depending on the wavenumber of the absorption band, A-type, B-type, and AB-type carbonation can be evidenced [1,18,44,64,65,66,67].

#### 3.2.1. FTIR Spectra of Hydroxyapatite and Strontium-Doped Hydroxyapatite Powders

The FTIR spectra of the HAp and HApSr powders are presented in Figure S4. In the FTIR spectrum of HAp powder (see Figure S4a), eight absorption bands were identified: 1091 cm^−1^ and 1029 cm^−1^, 963 cm^−1^, 871 cm^−1^, 633 cm^−1^, 601 cm^−1^ and 563 cm^−1^, and 473 cm^−1^, characteristic for the asymmetric stretching mode of PO_4_^3−^ (ν_3_), symmetric stretching mode of PO_4_^3−^ (ν_1_), CO_3_^2−^ group, libration mode of OH^−^ (ν_L_), bending mode of PO_4_^3−^ (ν_4_), and bending mode of PO_4_^3−^ (ν_2_) [1]. For the HApSr powder, the FTIR spectrum indicates slight shifts in the absorption bands (see Figure S4b). The absorption bands at 871 cm^−1^ and 869 cm^−1^ from the HAp and HApSr FTIR spectra indicate B-type carbonation (PO_4_^3−^ sites are substituted by CO_3_^2−^) [68].

#### 3.2.2. FTIR Spectra of Hydroxyapatite and Strontium-Doped Hydroxyapatite Coatings

**Figure 4:**
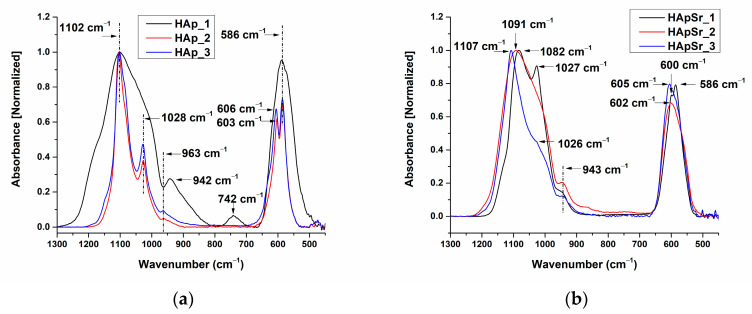
FTIR spectra of the HAp (**a**) and HApSr (**b**) coatings deposited on Ti substrates at different substrate temperatures.

The FTIR spectra of HAp and HApSr coatings deposited on optically polished Ti substrates by RF-MS, at different substrate temperatures, are presented in Figure 4a,b.

In the 1300–800 cm^−1^ wavenumber range, three absorption bands were identified for the HAp coatings, namely as follows: at ~1102 cm^−1^ and ~1028 cm^−1^, assigned to the asymmetric stretching mode of P-O (ν_3_) [45,69]; and at 942 cm^−1^ (HAp_1), respectively, ~963 cm^−1^ (HAp_2 and HAp_3), characteristic of the symmetric stretching mode of P-O (ν_1_) [45,69] (see Figure 4). In the FTIR spectrum of HAp_1 (see Figure 4a, black line), the broadening of the absorption band can be observed when compared to the absorption bands of HAp_2 (see Figure 4a, red line) and HAp_3 (see Figure 4a, blue line). Broad absorption bands in an FTIR spectrum may suggest the presence of amorphous calcium phosphates. The absorption bands characteristic of ν_3_ and ν_4_ vibrational modes in the FTIR spectra of HAp_2 and HAp_3 (see Figure 4a, red and blue line) are narrower and split, indicating the crystallization onset of the deposited layers [70].

Uskoković [70] evidenced by FTIR spectroscopy, the different crystallization states of amorphous calcium phosphates (ACP) annealed at different temperatures ranging between 100 and 1000 °C. The crystallization of amorphous calcium phosphates at 800 °C was shown by the narrowing of the ν_3_ band (P-O) and a splitting of the ν_4_ band (O-P-O) in the FTIR spectra and was confirmed by XRD analysis.

In the wavenumber range of 700–450 cm^−1^, the absorption band characteristic to ν_4_ bending mode of PO_4_^3−^ in the HAp_1 FTIR spectrum is centered at 587 cm^−1^, while for the HAp_2 and HAp_3 spectra, a split of the absorption band can be observed at 606 cm^−1^ (HAp_3), 603 cm^−1^ (HAp_2), and 586 cm^−1^ (see Figure 4a). We suppose that the position of the IR bands characteristic of ν_4_ bending mode of PO_4_^3−^ at 586 cm^−1^ may indicate that dehydroxylation occurs due to the substrate heating during plasma deposition. Pluduma [71] et al. studied the dependency between the OH^−^ percentage and the shifts of the IR bands in the 700–500 cm^−1^ wavenumber range. The deconvoluted peaks at 602 cm^−1^ and 575 cm^−1^, obtained for 100% OH^−^ amount were shifted to 604 cm^−1^ and 583 cm^−1^ for 8 ± 1% OH^−^ amount.

For the HApSr coatings (Figure 4b), three absorption bands were identified in the FTIR spectra in the 1300–800 cm^−1^ wavenumber range, namely 1082 cm^−1^ (HApSr_1), 1091 cm^−1^ (HApSr_2), 1107 cm^−1^ (HApSr_3), and ~1027 cm^−1^ (HApSr_1 and HApSr_3), characteristic of the asymmetric stretching mode of P-O (ν_3_) [69]; at 943 cm^−1^, characteristic of the symmetric stretching mode of P-O (ν_1_) [69]. Li [69] previously reported that the Sr doping of HAp structure can lead to shifts of the 963 cm^−1^ IR band to 943 cm^−1^.

#### 3.2.3. FTIR Spectra of Hydroxyapatite/Chitosan and Strontium-Doped Hydroxyapatite/Chitosan Coatings

**Figure 5:**
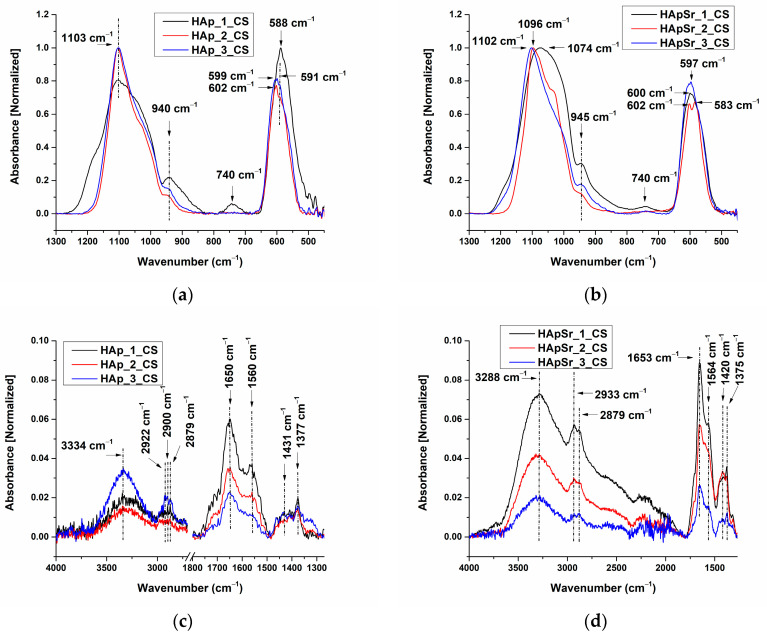
FTIR spectra of the HAp_CS (**a**,**c**) and HApSr_CS (**b**,**d**) coatings deposited on Ti substrates at different substrate temperatures.

The FTIR spectra of HAp_CS and HApSr_CS coatings deposited on Ti substrates with optically polished surfaces 1300–450 cm^−1^ and 4000–1300 cm^−1^ wavenumber ranges are presented in Figure 5a,b and Figure 5c,d, respectively.

##### FTIR Spectra of HAp_CS Samples

The absorption band characteristics of asymmetric and symmetric stretching modes of P-O bonds were identified in the FTIR spectra of the HAp_CS coatings at 1103 cm^−1^ and 940 cm^−1^ (see Figure 5a). When compared to the HAp coatings (see Figure 4a), the shifts from ~1102 cm^−1^ to 1103 cm^−1^ and from 942 cm^−1^ to 940 cm^−1^ are not significant, considering the FTIR resolution of 4 cm^−1^. However, for HAp_2, HAp_3 and HAp_2_CS and HAp_3_CS, the absorption bands from 963 cm^−1^ (see Figure 4a) were shifted to 940 cm^−1^ (see Figure 5a). This shift may be associated with the chitosan deposition process and consecutively to the overlapping of P-O and C-O IR bands, as chitosan has an absorption band around 942 cm^−1^, assigned to the stretching vibration of C-O [18]. The broadening of the absorption bands in the 1300–800 cm^−1^ wavenumber range may also be associated with the chitosan deposition process, considering that chitosan has absorption bands at 1059, 1027, and 990 cm^−1^, characteristic of the symmetric stretching of C-O in C-OH, C-O-C, and CH_2_OH groups [18].

In the 700–450 cm^−1^ wavenumber range, the IR band from 588 cm^−1^ (in the IR spectrum of HAp_1_CS—Figure 5a, black line) is slightly shifted from 586 cm^−1^ (in the IR spectrum of HAp_1—Figure 4a). There are shifts in the position of the IR bands characteristic of HAp_2_CS and HAp_3_CS samples when the substrate temperature increases (see Figure 5), accompanied by their narrowing.

##### FTIR Spectra of HApSr_CS Samples

In the FTIR spectra of the HApSr, HApSr_CS samples deposited at different substrate temperatures the absorption bands from 1107 cm^−1^, 1091 cm^−1^, 1082 cm^−1^ were shifted to 1102 cm^−1^, 1096 cm^−1^, and 1074 cm^−1^, respectively. In the wavenumber range of 700–450 cm^−1^, the absorption bands of the HApSr_1_CS and HApSr_3_CS spectra were centered at 600 cm^−1^ and 597 cm^−1^, respectively. Splitting of this band was observed in the HApSr_2_CS spectrum (602 and 583 cm^−1^).

In the wavenumber range of 4000–1300 cm^−1^ (Figure 5c,d), absorption bands characteristic for chitosan were identified, as follows: 3650–3000 cm^−1^ with maxima at 3334 cm^−1^, 2922 cm^−1^ and 2879 cm^−1^, 1650 cm^−1^, 1560 cm^−1^, 1431 cm^−1^, 1377 cm^−1^ for the HAp_CS coatings and 3650–3000 cm^−1^ with maxima at 3288 cm^−1^, 2933 cm^−1^ and 2879 cm^−1^, 1653 cm^−1^, 1564 cm^−1^, 1420 cm^−1^, and 1375 cm^−1^ for the HApSr_CS coatings. These bands are characteristic of the following: polymeric O-H stretch or N-H [72], asymmetric stretching of CH_2_ and symmetric stretching of -CH_3_ [72], stretching of (-C=O-) of amide I group or N-H stretching of amide I [44], bending of N-H in amide II [72], asymmetric bending of C-H in (-CH_3_) [72], and symmetric bending of C-H in (-CH_3_) [72], respectively.

**Figure 6:**
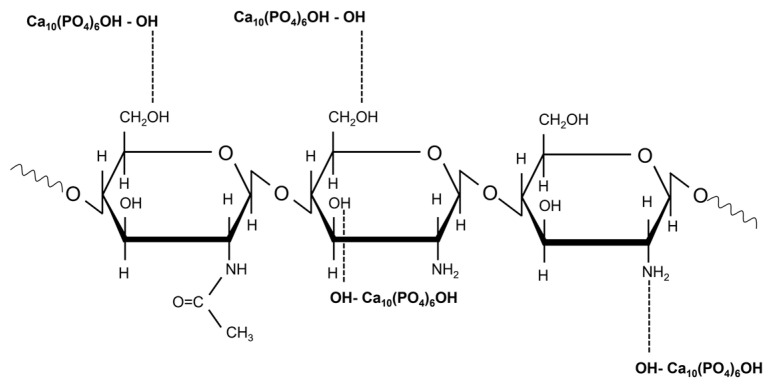
Graphical representation of HAp-CS interaction.

By correlating the information given by the XPS (see Table 2) and the FTIR spectra the incorporation of CS in Hap, respectively, HApSr layers occurs at least in a thin layer of about a few nanometers. Several previous results [73,74] showed that the chemical linkage between CS and HAp (respectively, HApSr) occurred by hydrogen bonds being formed between the hydroxyl (-OH) group of HAp and the hydroxyl or amine group (-NH_2_) of CS (see Figure 6). The -OH and -NH_2_ groups are end groups in the chemical structure of CS and HAp. Their chemical interaction is manifested by disappearance or deformation of ether bond in the pyranose ring from ~1150 cm^−1^ or of ~1250 cm^−1^ IR band characteristic of the amide III group [73]. In the spectra from Figure 5 these bands are overlapped with P-O bands and their absence or presence can be revealed only by peak fitting analysis. In our previous study [18] we performed such analysis in the 1200–800 cm^−1^ range in the case of CaP-CS layers generated by RF-MS and MAPLE techniques and we did not identify any of them [18].

### 3.3. Energy Dispersive X-ray Spectroscopy

**Figure 7:**
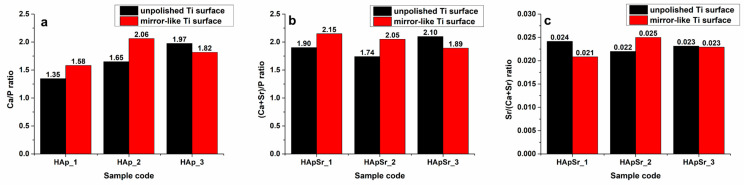
EDX results: Ca/P ratio for HAp layers (**a**), (Ca + Sr)/P (**b**) and Sr/(Ca + Sr) ratio (**c**) for HApSr layers deposited by RF-MS at different substrate temperatures.

The elemental composition of the HAp and HApSr coatings on unpolished and mirror like Ti surfaces was evaluated by EDX investigation. The Ca/P ratio (see Figure 7a) and (Ca + Sr)/P ratio (see Figure 7b) were calculated.

For the HAp coatings deposited on unpolished Ti surfaces (see Figure 7a, black columns), the Ca/P ratio increases from 1.35 (HAp_1) to 1.97 (HAp_3), as a function of the substrate temperature increase from 25 to 400 °C. For the HApSr_1 coating (see Figure 7b, black columns), the (Ca + Sr)/P ratio is higher compared to the Ca/P ratio of the HAp_1 coating deposited at the same substrate temperature (see Figure 7a, black columns).

A similar dependence on the substrate temperature was observed for the Ca/P (see Figure 7a red columns) and (Ca + Sr)/P ratios (see Figure 7b, red columns) that correspond to the layers deposited on mirror-like Ti surfaces. The Ca/P and (Ca + Sr)/P ratios of 1.65 and 1.74, respectively, the closest values to 1.67 (characteristic to stochiometric HAp), were obtained for the HAp_2 and HApSr_2 layers deposited on unpolished Ti substrates.

The Sr/(Ca + Sr) ratios (see Figure 7c) have similar values for all the samples.

The increase of Ca/P ratio can be related to the RF-MS deposition conditions, namely the re-sputtering of the phosphorous atoms from the growing layer due to oxygen negative ion bombardment [75]. On the other hand, the increase of Ca/P ratio with the substrate temperature can indicate the oxidation of the growing layer and the formation of CaO [18,75]. The fluctuation in the values of (Ca + Sr)/P and Ca/P ratios seems to be related to the substrate characteristics. In our previous study [44], in which the same RF-MS deposition conditions were applied for the generation of Sr-doped CaPs layers, but on Si substrates with mirror like surfaces, the (Ca + Sr)/P ratios were not affected by the substrate temperatures.

### 3.4. Scanning Electron Microscpoy

#### 3.4.1. Hydroxyapatite and Strontium-Doped Hydroxyapatite Coatings

**Figure 8:**
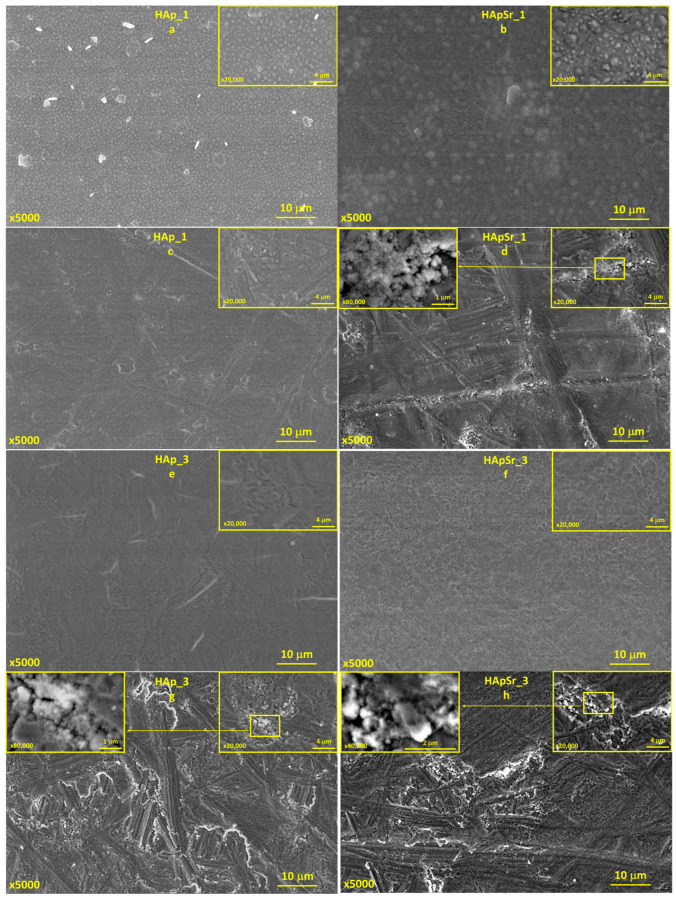
SEM images of HAp and HApSr coatings deposited at different substrate temperatures on mirror like (**a**,**b**,**e**,**f**) and unpolished Ti substrates (**c**,**d**,**g**,**h**).

The morphology of the HAp and HApSr coatings deposited by RF-MS on mirror-like and unpolished Ti substrate surfaces are presented in Figure 8 and Figure S5. The SEM investigation revealed that the substrate morphology is strongly dependent on the substrate temperature during the RF-MS deposition and on the surface type (mirror-like or unpolished).

For the coatings deposited on mirror-like Ti surfaces and on the Ti unpolished one, at a substrate temperature of 25 °C, a grain-like morphology was evidenced (see Figure 8a–d). The grains have quasi-spherical shapes, with smaller sizes for the HAp_1 sample (see Figure 8a, yellow border enclosure) compared to the HApSr_1 (see Figure 8b, yellow border enclosure).

In the case of unpolished substrates, the grains are even smaller (see Figure 8c,d, yellow borders), probably due to the fact that the unpolished surface ensures more nucleation sites for HAp and HApSr growth.

At the substrate temperature of 100 °C, the morphology of the HAp and HApSr coating surfaces changes, indicating combined grain-like and microchannel structures (see Figure S5a). These microchannels are formed most probably due to the coalescence of the grains [44] as the substrate temperature is increased. On the unpolished Ti substrates, only grain-like structures are formed (see Figure S5b,d).

As the substrate temperature was further increased up to 400 °C, the grain-like morphology of the HAp and HApSr coatings deposited on mirror-like (see Figure 8e,f) and unpolished (see Figure 8g,h) Ti surfaces completely disappeared.

The HAp_3 coating deposited on Ti with a mirror-like surface (see Figure 8e) presents compact microchannel structures (when compared to the HAp_2 coating—see Figure S5a), meaning that an increase of 300 °C (from 100 °C to 400 °C) in the substrate temperature significantly impacts the layer surface morphology. For the HApSr_3 (see Figure 8f) the microchannels are dense and uniformly distributed on the entire sample surface. In the case of the surfaces of HAp and HApSr layers deposited on unpolished Ti substrates, the grain-like structures compact (see Figure 8g,h yellow border enclosures).

#### 3.4.2. Hydroxyapatite/Chitosan and Strontium-Doped Hydroxyapatite/Chitosan Coatings

**Figure 9:**
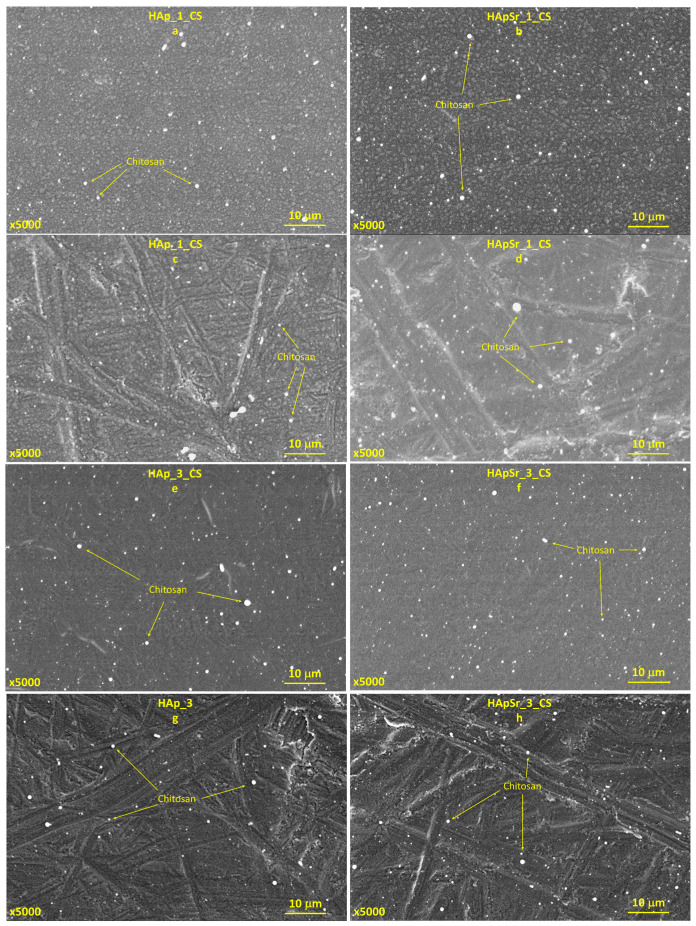
SEM images of HAp_CS and HApSr_CS coatings deposited at different substrate temperatures on mirror like (**a**,**b**,**e**,**f**) and unpolished Ti substrates (**c**,**d**,**g**,**h**).

The morphology of the HAp_CS and HApSr_CS coatings on mirror-like and unpolished Ti samples are presented in Figure 9 and Figure S6. When comparing them with the HAp and HApSr layers (see Figure 8 and Figure S5), the morphology is preserved. New spherical structures were identified on the analyzed surfaces, which, based on the EDX results (increased C and N content), were associated with CS. The grain and microchannel boundaries are not well defined, leading to the assumption that CS completely covered the HAp and HApSr layers in a film form.

Even if the morphologies of all the layers deposited on Ti substrates with mirror-like surfaces are similar to the ones generated on Si substrates [44], these morphological studies are of direct interest from the biological point of view, as Ti is the most used material for orthopedic implants. Moreover, for Ti substrates, blistering of the layers was not observed, probably due to proper matching between the thermal conductivity of the Ti substrate and the deposited layers.

### 3.5. Roughness Measurements

The R_a_ roughness parameters of the HApSr and HApSr_CS sample surfaces were measured and are presented in Table 3. The R_a_ of the layer surfaces deposited on optical polished Ti substrates increases as the temperature of the substrate during the RF-MS deposition is increased. In the case of layers deposited on unpolished Ti substrates the R_a_ parameters have random values.

These results suggest that the roughness of the Ti substrate surface does not influence the biological properties of the layers, considering that all the MTT, LDH, “live/dead”, and antimicrobial assays were conducted on the unpolished ones. The HApSr_2_CS and HApSr_3_CS represent the samples with the best cell viability, cytotoxicity, and antimicrobial activity properties, and have potential for bone tissue engineering.

### 3.6. Biological Assays

The biological properties were evaluated in order to determine the potential of the composite layers for bone tissue engineering applications. In this regard, the MTT, LDH, and live/dead assays, along with the antimicrobial activity against *S. aureus* were conducted for the HAp, HApSr, HAp_CS, and HApSr_CS layers deposited on unpolished Ti substrates.

#### 3.6.1. MTT Assay

**Figure 10:**
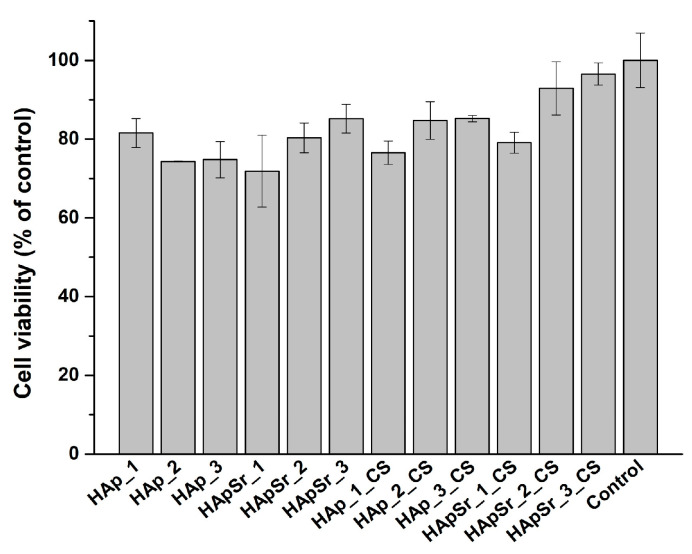
MTT cell viability assay of L929 cells on HAp, HApSr, HAp_CS, and HApSr_CS layers deposited under different experimental conditions in comparison with control—unstimulated cells, standard conditions.

The MTT assay (see Figure 10) was required to evaluate the cell viability (L929 cell line) in the presence of HAp and HApSr coatings, deposited by the RF-MS technique and of the HAp_CS and HApSr_CS coatings, deposited by RF-MS and MAPLE techniques, compared to the control (unstimulated cells). All deposited coatings were biocompatible. However, differences in the cell viability were observed as a function of the substrate temperature, the Sr content in HAp, and the CS presence.

The MTT results revealed that the best cell viability was achieved for the HApSr_2_CS (92.9% ± 6.8%) and HApSr_3_CS (96.5% ± 2.8%) coatings compared to the control (100% ± 6.9%). The corresponding coatings without chitosan—HApSr_2 and HApSr_3—had lower cell viability: 80.3% ± 3.8% and 85.2% ± 3.6%, respectively. The cell viability percentages for HAp_2_CS and Hap_3_CS are higher than those of the corresponding coatings without CS: HAp_2 and HAp_3. These findings reveal the advantage of CS deposition to improvement of the biocompatibility of the HApSr and HAp coatings.

By comparing the HAp_2_CS and HAp_3_CS samples with the HApSr_2_CS and HApSr_3_CS samples, the contribution of the Sr doping of HAp structure to the increase of cell viability is also shown.

Therefore, the addition of both Sr and CS to the HAp layers improves the cell viability for HApSr_2_CS and HApSr_3_CS.

Added separately to HAp coatings, Sr [76] or CS [77] improved the viability of L929 cells. The reason for improved L929 cell viability in the presence of Sr is related to the promotion of cell proliferation [78]. It has the same contribution to osteoblast cells while osteoclast cells have an inhibitory activity through a different biological mechanisms [14,79].

#### 3.6.2. LDH Assay

**Figure 11:**
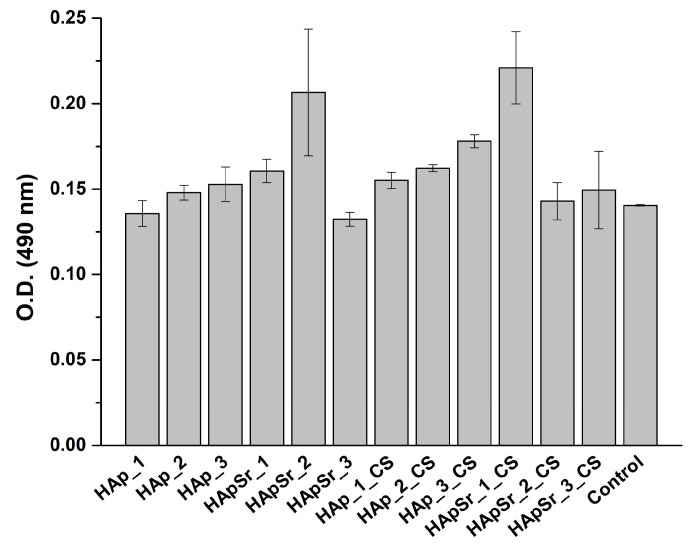
LDH cytotoxicity assay of L929 cells on HAp, HApSr, HAp_CS, and HApSr_CS layers deposited under different experimental conditions in comparison with control—unstimulated cells, standard conditions.

The LDH assay (see Figure 11) was required to evaluate the cell cytotoxicity (L929 cell line) in the presence of HAp and HApSr coatings, deposited by the RF-MS technique and of the HAp_CS and HApSr_CS coatings, deposited by RF-MS and MAPLE techniques, compared to the control (unstimulated cells).

The HApSr_2 and HApSr_1_CS coatings, O.D._490nm_ values of 0.21 ± 0.04 and 0.22 ± 0.02, respectively, had the highest level of toxicity compared to the control: O.D._490nm_ = 0.14.

The HApSr_2_CS and HApSr_3_CS had low cytotoxicity, with O.D._490nm_ values of 0.14 ± 0.01 and 0.15 ± 0.02, respectively.

Considering the results of the biocompatibility assays, namely the MTT and LDH tests, the HApSr_2_CS and HApSr_3_CS exhibited the highest degree of biocompatibility considering the high cell proliferation (see Figure 10) and the low toxicity (see Figure 11).

#### 3.6.3. Live/Dead Assay

**Figure 12:**
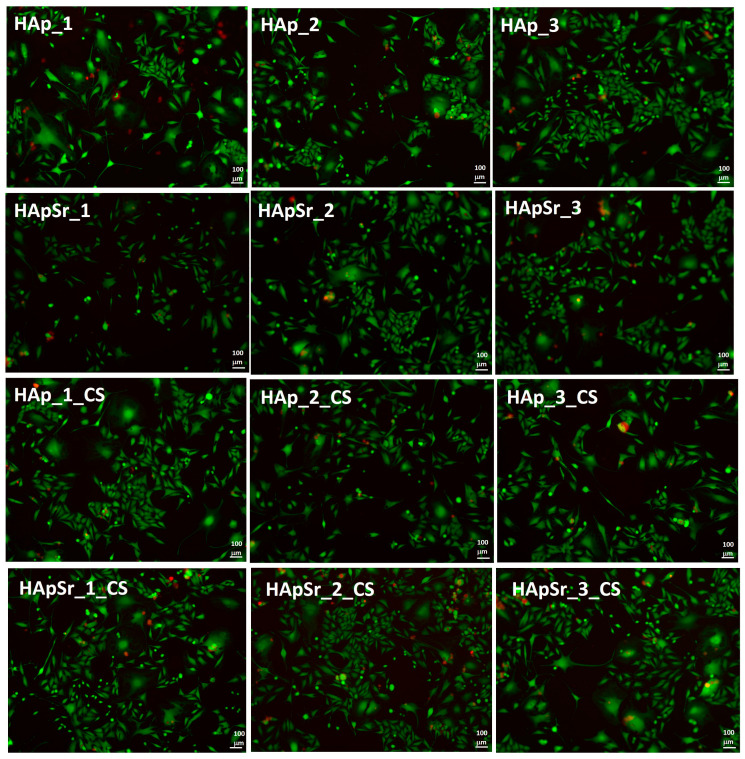
Fluorescence microscopy images of “live/dead” cell assay of L929 cells on HAp, HApSr, HAp_CS, and HApSr_CS layers deposited under different experimental conditions.

The cell viability was also evaluated by the live/dead cell assay of L929 cells in the presence of the layers. The fluorescence microscopy images are presented in Figure 12 and Figure S7 (control, unstimulated cells).

The live/dead viability test showed that all composites were biocompatible. The majority of cells grown on the composites were viable (green color) regardless of the coating type.

#### 3.6.4. Antimicrobial Activity

**Figure 13:**
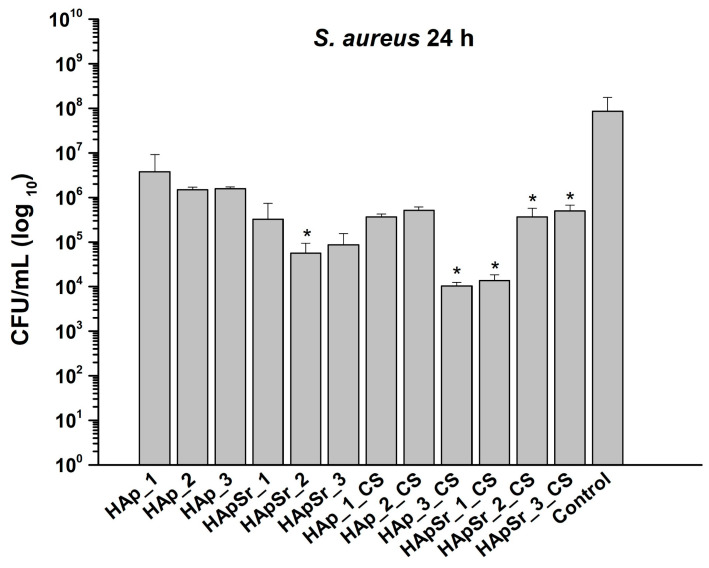
Testing of antimicrobial activity against *Staphylococcus aureus* ATCC strain * *p* < 0.05, one way ANOVA post hoc Bonferroni test; control—bacterial culture, standard conditions.

The antimicrobial activity against *S. aureus* is presented in Figure 13.

All tested materials showed a degree of antimicrobial activity against *S. aureus*. The highest level of activity was observed in the case of HApSr_1-3_CS materials, and also in the case of HAp_3_CS.

O’Sullivan et al. [80] deposited Ag and Sr-doped HAp coatings on Ti by the CoBlast technique and evaluated the biofilm inhibition against *S. aureus*. Better results in preventing microbial attachment were observed for Ag doped HAp then for Sr or Ag-Sr doped HAp compared to HAp. However, when testing the metabolic activity of MG-63 cells in the presence of the coatings, the Sr-doped HAp statistically improved the metabolic activity.

## 4. Conclusions

The results presented in this study link the biological properties of HAp, HApSr, HAp_CS, and HApSr_CS layers deposited on Ti substrates by plasma and laser techniques under different experimental conditions, with their physicochemical properties. All the samples have biocompatible properties and antimicrobial activity against *Staphylococcus aureus*. A temperature at the substrate of 400 °C, maintained during the entire duration of the radio-frequency magnetron sputtering depositions, favors the generation of HApSr layers, which, by chitosan covering, increased the biocompatibility and antibacterial properties. Fluorescence microscopy indicated a minimal dead number of cells for all the analyzed samples.

The morphology of HAp and HApSr layers was better evidenced when they were deposited on optically polished substrate surfaces and was of grain-like and microchannel structures when the substrate temperature was 25 °C and 400 °C, respectively.

The apatite structure at the surface of HApSr_CS samples was evidenced by the Ca 2p3/2 XPS peak observed at HApSr_1_CS, HApSr_2_CS and HApSr_3_CS sample surfaces. For all the analyzed samples, the FTIR studies indicated shifts in the position of P-O absorption bands as a function of the deposition conditions, the Sr or CS content, as well as the substrate type [44].

## Figures and Tables

**Table 1:** XPS binding energies (eV) assignment for the HAp and HApSr coatings.

XPS Peaks	Binding Energy (eV)						XPS Peak Assignment
	HAp_1	HAp_2	HAp_3	HApSr_1	HApSr_2	HApSr_3	
**Ca 2p_1/2_**	350.6	350.5	350.5	350.3	350.7	350.5	Ca-Ca; Ca-OH; Ca-O in HAp [20,54,55]
	351.5	351.4	351.2	351.1	351.3	351.2	Ca-O in a calcium phosphate phase [20]
**Ca 2p_3/2_**	347.1	347	347	346.8	347.2	347	Ca-Ca; Ca-OH; Ca-O in HAp [20,54,55,56]
	348	347.9	347.7	347.6	347.8	347.7	Ca-O in a calcium phosphate phase [20]
**P 2p_1/2_**	134	133.9	133.8	133.8	134.1	133.9	P-O in PO_4_ of Hap [20,54,55]
**P 2p_3/2_**	133.2	133.1	132.9	132.9	133.3	133	P-O in PO_4_ of Hap [20,54,55]
**O 1s**	532.2	532.4	532.5	532.3	532.5	532.7	HPO_4_^2−^ [18]
	531.3	531.5	531.3	531.1	531.3	531.3	O-P, O-H, and O-C in PO_4_^3−^, OH^−^ and CO_3_^2−^ [20]
	530.7	530.8	530.7	530.5	530.6	530.6	O-Ca in Hap and CaCO_3_ [20,54]
**C 1s**	288.6	288.4	288.5	288.5	288.6	288.4	O-C=O/CO_3_^2−^ [52]
	287.4	287.4	287.4	287.4	287.4	287.4	C=O [52]
	286	286	286	286	286.1	286.1	C-OH/C-O-C [52]
	284.6	284.6	284.6	284.6	284.6	284.6	C-C/C-H [52,57,58]

**Table 3:** Roughness measurements of the HApSr and HApSr_CS layers surfaces deposited under different experimental conditions.

Sample Code	R_a_ (nm) Optically Polished/Unpolished
HApSr_1	27/541
HApSr_2	32/360
HApSr_3	47/384
HApSr_1_CS	27/381
HApSr_2_CS	38/403
HApSr_3_CS	49/376

## Data Availability

The original contributions presented in the study are included in the article/Supplementary Materials, further inquiries can be directed to the corresponding author/s.

## Supplementary Materials

The following supporting information can be downloaded at: https://www.mdpi.com/article/10.3390/polym16131922/s1, Figure S1: XPS survey spectra of HAp and HApSr coatings; Figure S2: XPS high-resolution deconvoluted spectra of HAp_1: (a) Ca 2p, (b) P 2p, (c) O 1s, and (d) C 1s and HAp_1_CS: (e) Ca 2p, (f) N 1s, (g) O 1s, and (h) C 1s; Figure S3: XPS survey spectra of HAp_CS and HApSr_CS coatings; Figure S4: FTIR spectra of the HAp (a) and HApSr (b) powders obtained by Microwave-assisted Hydrothermal synthesis in the wavenumber range 1350–450 cm^−1^; Figure S5: SEM images of HAp_2 (a and b) and HApSr_2 (c and d) coatings deposited on mirror-like (a and c) and unpolished Ti (b and d) samples at a substrate temperature of 100 °C; Figure S6: SEM images of HAp_2_CS (a and b) and HApSr_2_CS (c and d) coatings deposited on mirror-like (a and c) and unpolished Ti (b and d) samples at a substrate temperature of 100 °C; Figure S7: Fluorescence microscopy image of “Live/Dead” cell assay of L929 cells—control, standard conditions.
