# Supplementary material for: Biological and Physicochemical Analysis of Sr-Doped Hydroxyapatite/Chitosan Composite Layers
Source: Polymers (Basel). 2024 Jul 5;16(13):1922. doi: 10.3390/polym16131922 (PMC11244040; doi:10.3390/polym16131922)
Supplement: Supplementary file 1 [file polymers-16-01922-s001.zip › polymers-3002570-supplementary.pdf]

polymers

MDPI

*Supplementary Materials*

# Biological and Physicochemical Analysis of Sr-Doped Hydroxyapatite/Chitosan Composite Layers

**Maria Elena Zarif [1,2], Bogdan Bita [1,3], Sasa Alexandra Yehia-Alexe [1,3], Irina Negut [1], Gratiela Gradisteanu Pircalabioru [4,5,6], Ecaterina Andronescu [2,7,8] and Andreea Groza [1,*]**

1 National Institute for Lasers, Plasma and Radiation Physics, 77125 Măgurele, Romania; maria.zarif@inflpr.ro (M.E.Z.); bogdan.bita@inflpr.ro (B.B.); sasa.yehia@inflpr.ro (S.A.Y.-A.); negut.irina@inflpr.ro (I.N.)
2 Faculty of Chemical Engineering and Biotechnologies, University Politehnica of Bucharest, 011061 Bucharest, Romania; ecaterina.andronescu@upb.ro
3 Faculty of Physics, University of Bucharest 77125 Măgurele, Romania
4 eBio-Hub Research Center, University Politehnica of Bucharest-CAMPUS, 6 Iuliu Maniu Boulevard, 061344 Bucharest, Romania; gratiela.gradisteanu@icub.unibuc.ro
5 Research Institute of the University of Bucharest (ICUB), University of Bucharest, 050657 Bucharest, Romania
6 Department of Microbiology and Immunology, Faculty of Biology, University of Bucharest, 050657 Bucharest, Romania
7 Academy of Romanian Scientists, 3 Ilfov Str., District 5, 050044 Bucharest, Romania
8 National Research Center for Micro and Nanomaterials, University Politehnica of Bucharest, 060042 Bucharest, Romania
* Correspondence: andreea.groza@inflpr.ro

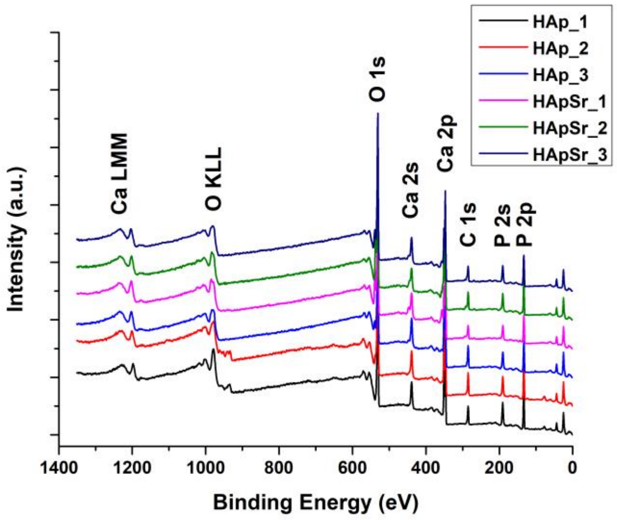


**Figure S1.** XPS survey spectra of HAp and HApSr coatings.

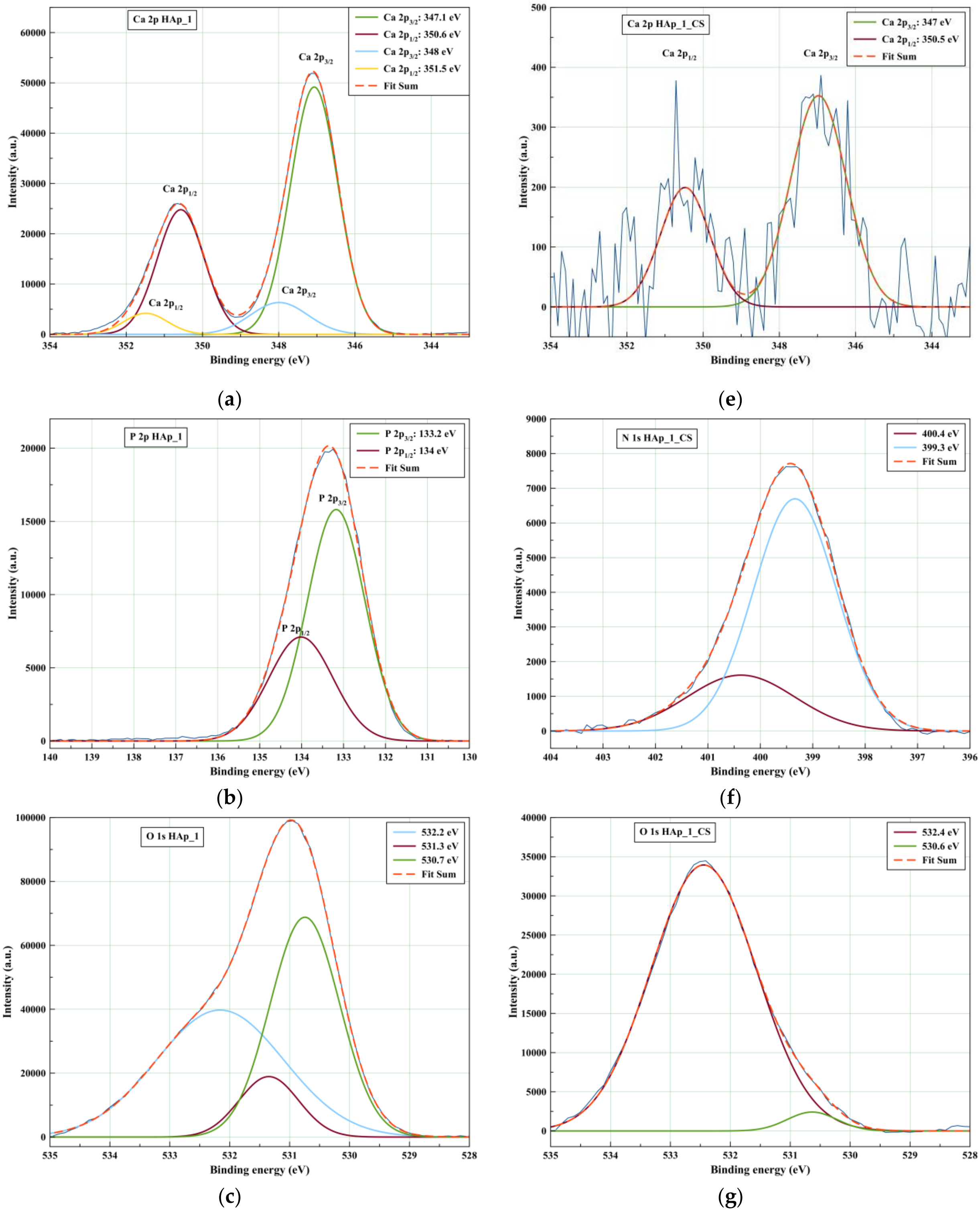

Ca 2p HAp_1
Ca 2p3/2: 347.1 eV
Ca 2p1/2: 350.6 eV
Ca 2p3/2: 348 eV
Ca 2p1/2: 351.5 eV
Fit Sum
Ca 2p3/2
Ca 2p1/2
Intensity (a.u.)
Binding energy (eV)
(a)
Ca 2p HAp_1_CS
Ca 2p3/2: 347 eV
Ca 2p1/2: 350.5 eV
Fit Sum
(e)
P 2p HAp_1
P 2p3/2: 133.2 eV
P 2p1/2: 134 eV
Fit Sum
P 2p3/2
P 2p1/2
(b)
N 1s HAp_1_CS
400.4 eV
399.3 eV
Fit Sum
(f)
O 1s HAp_1
532.2 eV
531.3 eV
530.7 eV
Fit Sum
(c)
O 1s HAp_1_CS
532.4 eV
530.6 eV
Fit Sum
(g)

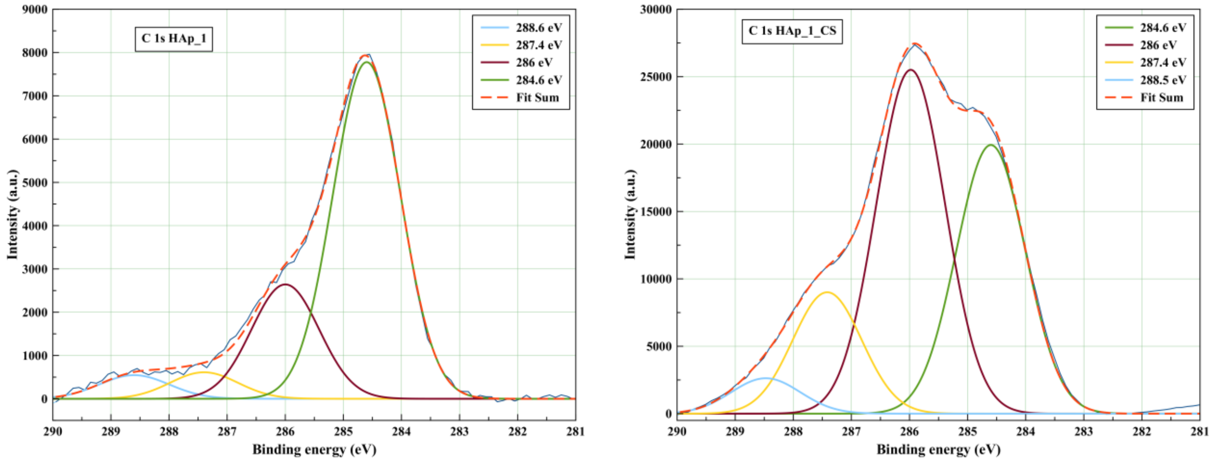


(**d**) (**h**)

**Figure S2.** XPS high-resolution deconvoluted spectra of HAp_1: (a) Ca 2p, (b) P 2p, (c) O 1s, and (d) C 1s and HAp_1_CS: (e) Ca 2p, (f) N 1s, (g) O 1s, and (h) C 1s.

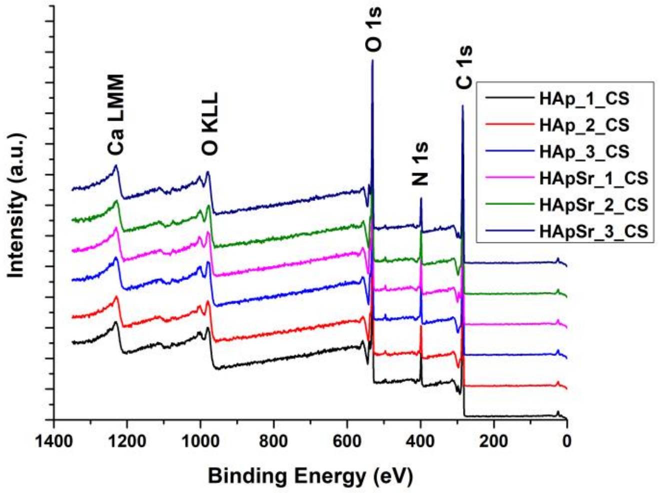


Figure **S3**. XPS survey spectra of HAp_CS and HApSr_CS coatings.

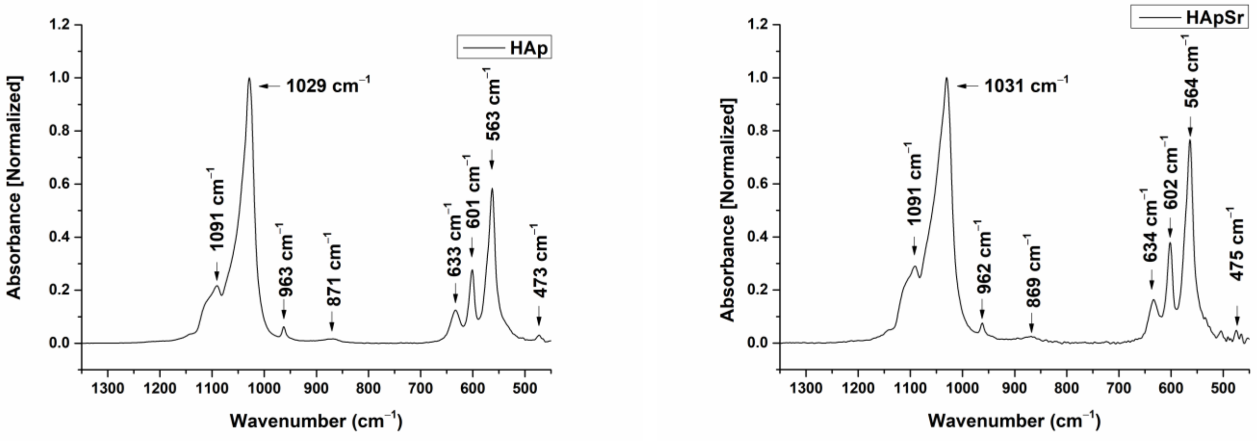


(**a**) (**b**)

**Figure S4.** FTIR spectra of the HAp (a) and HApSr (b) powders obtained by Microwave-assisted Hydrothermal synthesis in the wavenumber range 1350-450 $cm^{-1}$

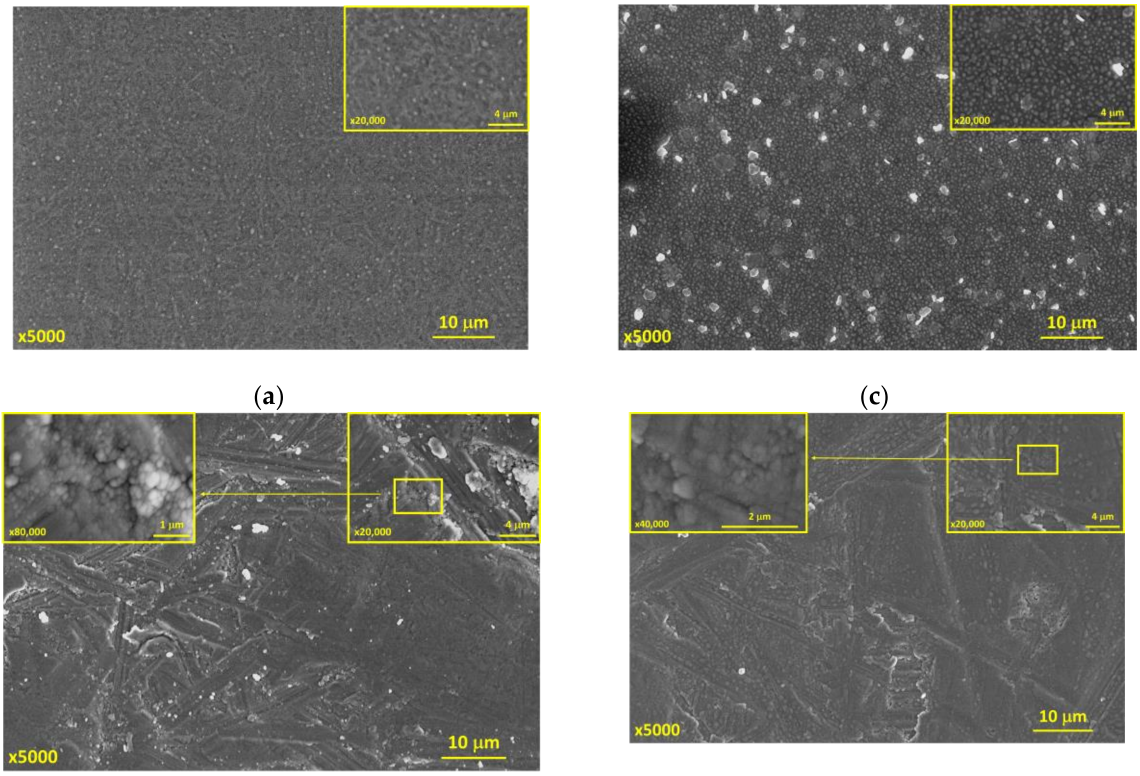


(**a**) (**c**)

(**b**) (**d**)

**Figure S5.** SEM images of HAp_2 (a and b) and HApSr_2 (c and d) coatings deposited on mirror-like (a and c) and unpolished Ti (b and d) samples at a substrate temperature of 100 °C.

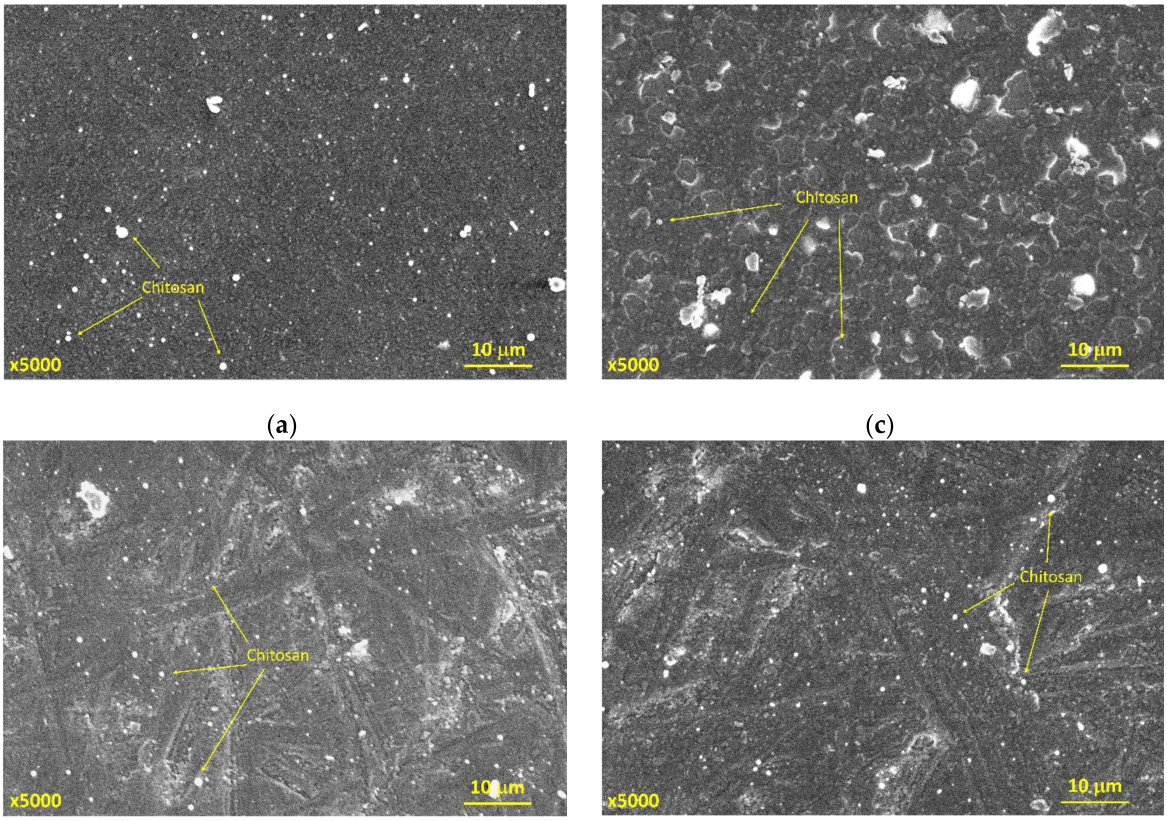


(**a**) (**c**)

(**b**) (**d**)

**Figure S6.** SEM images of HAp_2_CS (a and b) and HApSr_2_CS (c and d) coatings deposited on mirror-like (a and c) and unpolished Ti (b and d) samples at a substrate temperature of 100 °C.

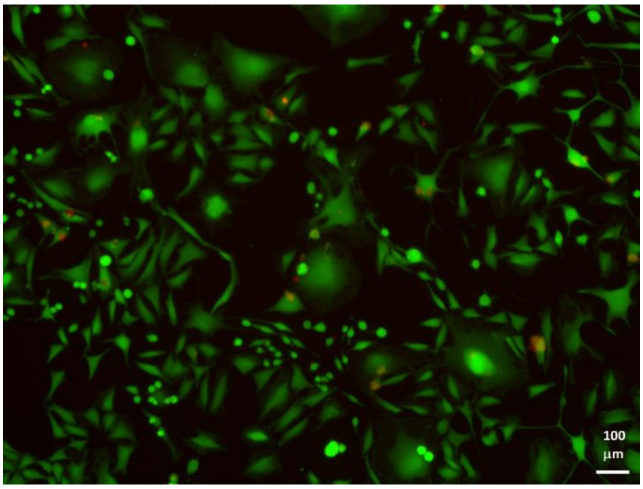


**Figure S7.** Fluorescence microscopy image of "Live/Dead" cell assay of L929 cells – control, standard conditions.
